# Novel nanocapsules with Co–TiC twin cores and regulable graphitic shells for superior electromagnetic wave absorption†

**DOI:** 10.1039/c8ra00040a

**Published:** 2018-02-08

**Authors:** Yuanliang Zhou, Javid Muhammad, Xuefeng Zhang, Dongxing Wang, Yuping Duan, Xinglong Dong, Zhidong Zhang

**Affiliations:** Key Laboratory of Materials Modification by Laser, Ion, and Electron Beams, School of Materials Science and Engineering, Dalian University of Technology Liaoning 116024 P. R. China dongxl@dlut.edu.cn; Key Laboratory for Anisotropy and Texture of Materials (MOE), School of Materials and Engineering, Northeastern University Shenyang 110819 P. R. China; Shenyang National Laboratory for Materials Science, Institute of Metal Research, Chinese Academy of Sciences Shenyang Liaoning 110015 P. R. China zdzhang@imr.ac.cn

## Abstract

The synthesis of nanometer materials with unique structures and compositions has proven successful towards the attenuation of electromagnetic (EM) waves. However, it is still a challenge to form special nanostructures by integrating magnetic/dielectric loss materials into one particle due to the difficulties in coupling the heterogeneous components. Herein, we present the synthesis of novel nanocapsules (NCs) with Co–TiC twin cores encapsulated inside graphitic shells using an arc-discharge plasma method. The thickness of the graphitic shell could be controlled by quantitatively tuning the carbon source concentration. The optimal reflection loss (RL) values of the prepared NCs was −66.59 dB at 8.76 GHz with a low thickness of 2.56 mm. The bandwidth of RL ≤ −10 dB was up to 14.4 GHz, which almost covered the entire frequency band, namely, the S to Ku band (3.6 GHz to 18 GHz). This superior EM wave absorption was ascribed to the specific double-core shell nanostructures and effective impedance matching between the magnetic loss and dielectric loss originating from the combination of the magnetic Co and dielectric TiC/C.

## Abstract 2

Fabrication of Co–TiC@C nanocapsules *via* an arc discharge plasma method. Its optimum EM wave absorption was −66.59 dB at 8.76 GHz.

## Graphical abstract

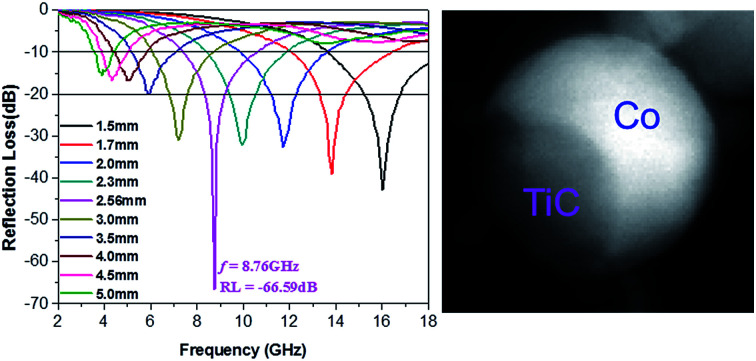

## 1. Introduction

With the wide application of wireless communication devices utilizing high-frequency EM waves as signal carriers, the speed and reliability of people's access to information have been greatly improved. Nevertheless, EM interference among adjacent signal lines is unavoidable. Over the past few years, considerable attention has been paid towards enhancing the EM wave absorbing properties of materials.^1–12^ Among the various kinds of absorbents, nanocapsules (NCs) comprising magnetic and dielectric components together, can promote the interface polarization and the multiple scattering of EM waves due to their large numbers of dangling bonds, core–shell synergistic effects and appropriate impedance matching.

Being an important magnetic material, Co not only has large anisotropic field and high saturation magnetization (168 emu g^−1^), but also has a Curie temperature that can reach up to 1404 K, which favors its use in elevated temperature environments when compared to iron (1043 K) and nickel (631 K). However, its characteristics of poor oxidation resistance, magnetic coupling and agglomeration, particularly at the nanoscale, may result in serious structural instability. To solve this dilemma, isolating the magnetic Co by dielectric phases has proven effective in recent articles. For example, porous 3D flower-like Co/CoO was synthesized using a two-step method comprised of a hydrothermal reaction and a subsequent annealing process, and its optimal RL value can reach −50 dB.^13^ Liu *et al.* investigated the EM absorption properties of Co/C nanoparticles with a low degree of graphitization and the results showed that the minimum RL value was −43.4 dB (16.8 GHz) at a thickness of 2.3 mm with an absorption bandwidth of 7.2 GHz (RL ≤ −20 dB).^14^ Luo *et al.* prepared cobalt-containing microwave absorption ceramic materials *via* the *in situ* pyrolysis of carbon-rich poly(dimethylsilylene)diacetylene with alkyls on the backbone coordinated with Co_2_(CO)_8_, which showed the minimum RL value was −42.43 dB at 10.55 GHz with an absorption bandwidth about 4 GHz (RL ≤ −10 dB).^15^ From the above results, it can be concluded that good EM wave absorption can be realized by constructing moderate microstructures of the cobalt-based composites. However, in these studies, most of them have focused on a single dielectric phase tuning the EM impedance matching. The EM wave absorbing mechanisms of dual dielectric phase coupled Co structures remain poorly understood.

Amongst the various dielectric materials, titanium carbide and carbon materials are prominent candidates with high prominence due to their vital roles in the dielectric loss of EM wave energies.^16–20^ Additionally, TiC has very low density (4.93 g cm^−3^), extreme hardness (28–35 GPa), high melting point (3340 K) and good electrical and thermal conductivities.^21,22^ While the tunable properties of carbon materials can be realized against oxidation and corrosion performances. To the best of our knowledge, research on the microwave absorption of composites with dual dielectric TiC/C materials coupled with magnetic Co have never been reported. Herein, the NCs of Co–TiC twin cores encapsulated inside graphitic shells were successfully prepared using an arc-discharge plasma method. By quantitatively controlling the gas ratios of CH_4_/Ar, it can effectively regulate the synergistic effect of the magnetic/dielectric losses and also provides the structural flexibility for an optimal RL value in the GHz range.

## 2. Experimental section

An arc-discharge plasma method was utilized to fabricate different kinds of NCs.^10,11^ In this work, coarse micro-sized Co and Ti powders in 300 meshes, used as the raw materials, were mixed uniformly in a mass ratio of 1 : 1, the mixture was compressed into a solid cylindrical block and then annealed in a vacuum oven at 500 °C for about 30 min to remove the adhered moisture and gaseous impurities. The annealed bulk acting as the anode of arc-discharge was laid on water-cooled copper stage, while a graphitic rod served as the opposite cathode. After the chamber was evacuated to 5.0 × 10^−3^ Pa, a mixed atmosphere of CH_4_ (carbon source) and Ar with different gas pressure ratios of 1 : 8, 1 : 4 and 1 : 2 were introduced to reach 22.5 kPa, 25 kPa and 30 kPa, respectively. The arc current was set at 90 A, while the voltage was controlled in range of 20–24 V depending on the distance between the two electrodes. Before collecting the resultant products, a self-passivation process was carried out for 8 h by introducing trace air into the working chamber. Accordingly, three samples A, B and C were prepared under the preparation conditions as shown in Table 1.

**Table 1:** Preparation conditions and yields of the carbon coated Co–TiC NCs

Sample	Gas pressure [kPa]		Arc voltage [V]	Arc current [A]	Yield [g h^−1^]
	CH_4_	Ar			
A	2.5	20	20	90	7.8
B	5	20	22	90	18.3
C	10	20	24	90	32.7

The as-prepared NCs were characterized by TEM (Tecnai^2^ 20 S-TWIM), STEM (Tecnai G^2^ F30 TWIN), X-ray diffraction (XRD, PANalytical Empyrean) using Cu Kα radiation (*λ* = 1.5416 Å), Raman spectroscopy (InVia) with a laser excitation wavelength of 514.5 nm and vibrating sample magnetometer (VSM, JDM-13). The EM parameters were determined using an Agilent PNA N5222A vector network analyzer in the frequency range of 2–18 GHz.

## 3. Results and discussion

### 3.1 Microstructure characterizations

**Fig. 1:**
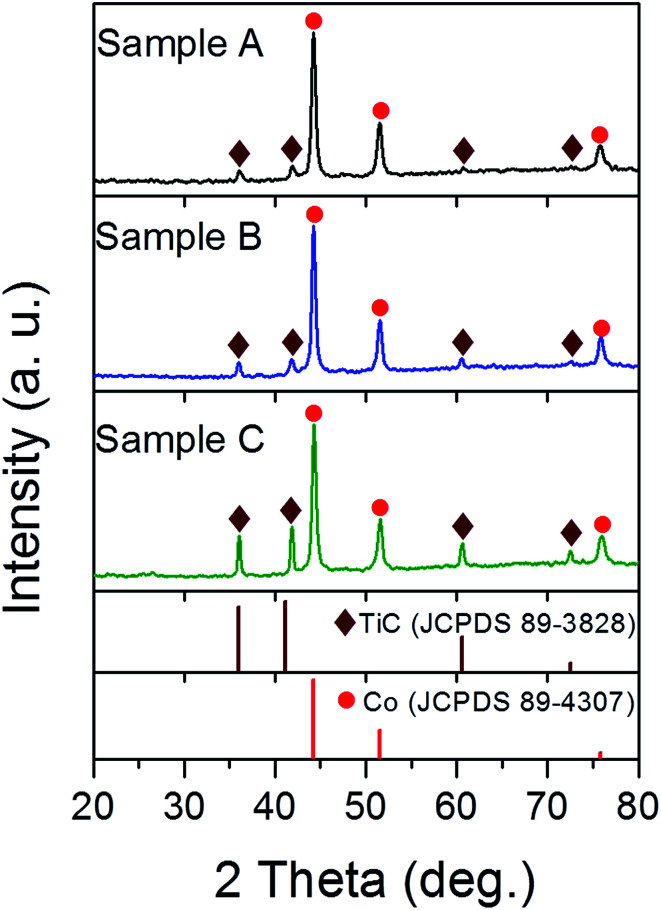
XRD patterns of samples A, B and C synthesised under a mixed atmosphere of CH_4_ and Ar with gas ratios of 1 : 8, 1 : 4 and 1 : 2, respectively.

Fig. 1 shows the XRD pattern of the three samples. The characteristic diffraction peaks at 44.2°, 51.5° and 75.8° were assigned to the (111), (200) and (220) lattice planes of fcc Co (JCPDS 89-4307), while the peaks at 35.99°, 41.8°, 60.6° and 72.5° belong to the (111), (200), (220) and (311) lattice planes of fcc TiC (JCPDS 89-3828), respectively. It is conceivable that the high temperature arc plasma can cause the pyrolysis of CH_4_ according to following equation: CH_4_ → C + 2H_2_ and then a chemical reaction continually occurs in the inner zone of the arc plasma: Ti + C → TiC. Meanwhile, the excessive carbon atoms undergo nucleation and growth to form the graphitic shells. The mass fractions of the Co and TiC phases were calculated from the XRD data using the reference intensity ratio (RIR) method^23^ and listed in Table S1.† The mass fraction of TiC in the as-made samples tends to increase upon enhancing the gas ratios of CH_4_/Ar from 1 : 8 to 1 : 2. The absence of any diffraction peaks from the graphitic shell was attributed to its thin layers, partially disordered state or no free carbonaceous phases in large sizes.

**Fig. 2:**
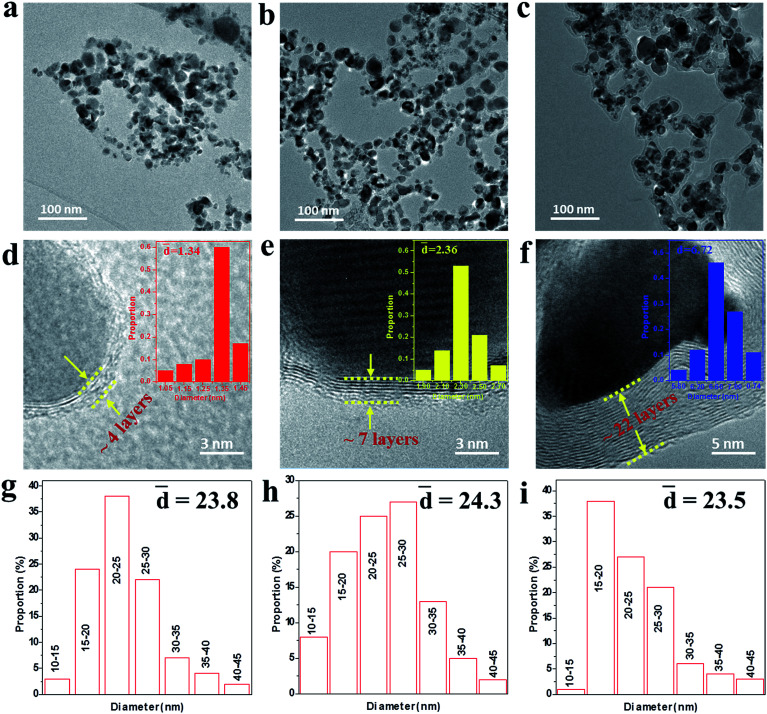
(a–c) TEM images of samples A, B and C, respectively. (d–f) The corresponding high-resolution TEM (HRTEM) images with the shell size distribution shown in the inset, indicating the thickness of the graphitic shell increases from sample A to C. (g–i) The particle size distribution of samples A–C.

**Fig. 3:**
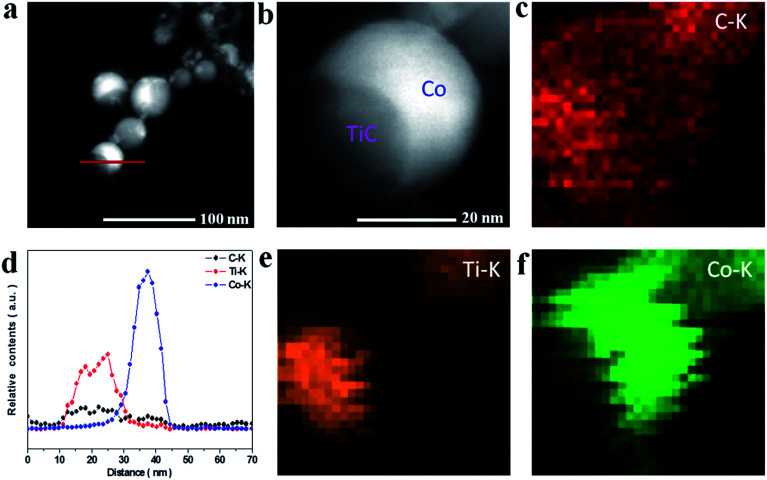
(a and b) HAADF images of sample B. (d) The line scanning elemental analysis marked in (a). (c, e and f) Elemental mapping images of C, Ti and Co.

To further reveal the microstructures of the as-made NCs, the TEM and HRTEM images were measured as shown in Fig. 2. It can be seen from Fig. 2a–c that the NCs present an irregular spherical shape ranging from 10 to 50 nm and the mean diameter was statistically calculated to be about 24 nm for samples A–C using ImageJ software, as shown in Fig. 2g–i. The HRTEM images under higher magnification (Fig. 2d–f) display the well-defined core@shell interfaces, indicating the features of the heterogeneous components in the NCs. The interplanar spacing of the graphitic layers was determined to be 0.34 nm, corresponding to the standard (002) plane of graphite. It is noteworthy that the number of graphitic layers increases from ∼4 to ∼22 upon increasing the amount of CH_4_ gas, which is consistent with the increasing trend of the statistical shell thickness. It was also found that the graphitic shells undergo an evolution from distorted thin layers to ordered multi-layers. Moreover, some obvious bending defects and ripples appeared in the shells, which can be attributed to the effects of the energetic Ar^+^/H^+^ ions in the plasma used in the preparation process. Furthermore, the three samples have similar microstructures and sample B was chosen as a typical example to demonstrate the structural details, as shown in Fig. 3. From the analysis of the line scanning images and elemental mapping presented in Fig. 3c–f, it can be concluded that the nanocapsule consists of a Co–TiC twin core and a graphitic shell, in which the graphitic shell thickness can be controllable by quantitatively tuning the concentration of methane gas.

**Fig. 4:**
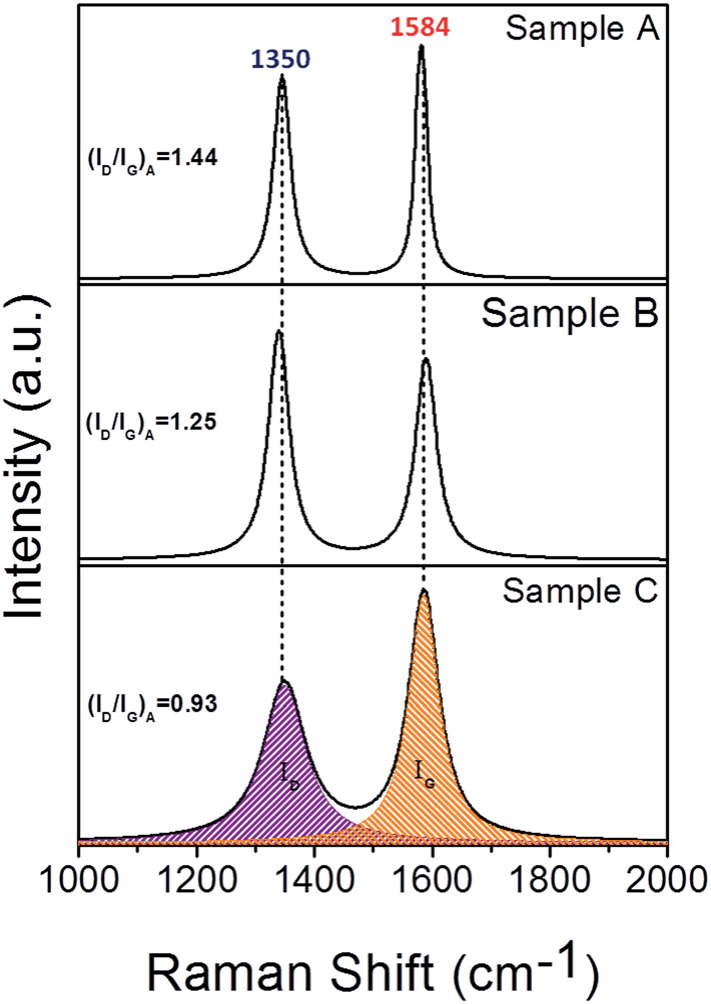
Raman spectra of samples A, B and C synthesised under a mixed atmosphere of CH_4_ and Ar with gas ratios of 1 : 8, 1 : 4 and 1 : 2, respectively.

In order to reveal the details of the graphitic shells in the Co–TiC@C NCs, the Raman spectra were measured at a scan step of 0.65 cm^−1^ as presented in Fig. 4. All the spectra have two intensive peaks around 1350 and 1584 cm^−1^, corresponding to the D peak (A_1g_ carbon vibration modes) and G peak (E_2g_ carbon vibration modes), respectively. The strong D peak is normally aroused by the presence of in-plane substitutional heteroatoms, vacancies, grain boundaries or other defects, meaning a large amount of disordered carbon originating from the stretching vibration of any C pairs of sp^2^ sites. With respect to the peak at 1580 cm^−1^ of bulk graphitic,^24^ the G peaks of the Co–TiC@C NCs samples shift to 1584 cm^−1^. Such a faint change in the G shifts may be caused by the introduction of topological disorder into the graphitic layers, where the C–C bonds are mainly attributed to sp^2^ hybridization that has been softened by weaker bonds.^25^ To some extent, the Raman intensity ratio of the D peak to G peak (*I*_D_/*I*_G_)_A_ can be used to evaluate the degree of graphitization. Here, the values of (*I*_D_/*I*_G_)_A_ were obtained from the area ratio of the D and G peaks after a Lorentzian function fitting of the Raman spectra and were determined to be 1.44, 1.25 and 0.93 for sample A, B and C, respectively. These values suggest that the disorder degree of the graphitic shells decreased. Generally, the value of (*I*_D_/*I*_G_)_A_ is inversely proportional to the in-plane correlation length (*L*_a_) if the size of the graphitic crystals exceeds 2 nm according to the Tuinstra–Koenig equation:^26^

1 *L*_a_ = *C*_λ_(*I*_D_/*I*_G_)^−1^_A_

where *C*_λ_ is about 4.4 nm, the calculated *L*_a_ was 3.05, 3.52 and 4.70 nm for samples A, B and C, respectively. The correlation lengths (*L*_a_) are considered to be proportional to the number of graphitic layers^26^ and are in agreement with the increased thickness of the graphitic shells in the Co–TiC@C NCs, shown in the Fig. 2d–f.

**Fig. 5:**
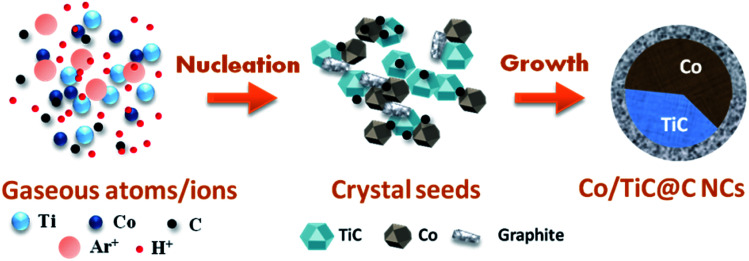
Schematic illustration of the formation process of the Co–TiC@C NCs.

Based on above experimental results and analysis, the formation mechanism for the Co–TiC@C NCs is schematically presented in Fig. 5. It is considered that three main steps are involved in this formation process: (1) the generation of gaseous atoms or ions from the raw species and assistant gases. The raw Co/Ti bulk is evaporated by the highly energetic plasma at temperatures over 10 000 K.^27^ At high temperature, H_2_ and Ar are separately ionized into H^+^ and Ar^+^, while CH_4_ is decomposed into C atoms and H^+^. These gaseous atoms/ions with certain concentrations and energy states in the initial formation stage will undergo condensation in the following steps. (2) The nucleation of crystal seeds. In this step, the Gibbs free energy (Δ*G*) of the chemical reactions or solidifications will play a dominant role in determining what kinds of seeds are formed, *e.g.* it is negative for the reaction between the Ti and C atoms, if temperature is higher than 1939 K, which favors the formation of TiC seeds.^28^ Large numbers of Co atoms will nucleate independently into pure Co seeds, while the carbon atoms also experience a similar nucleation process to form graphitic seeds. Under the high energy impact of the Ar^+^ ions and the inhibition effect of the H^+^ ions on graphitization, the two kinds of crystal seeds, *i.e.* TiC and Co clusters with the same crystal type (fcc) will be connected partly at the fresh surfaces and further combined into twin seeds through a coherent twin boundary. This kind of twin crystal Co–TiC will grow into one core of a nanocapsule. The existence of a certain crystallography orientation relationship between two seeds (both are fcc-crystal structures here) is crucial for the twin seeds. (3) Growth of the Co–TiC@C NCs. The tremendous temperature-gradient established between the arc zone and the inner wall of work chamber is the main cause of cooling and a necessary condition for species growth. The twin seeds of Co–TiC grow into larger ones *via* the absorption/diffusion of the Ti, C, Co species from the ambient atoms. Meanwhile the unreacted gaseous C atoms adhere to the surface of the twin seeds and subsequently grow into the graphitic shells. The higher CH_4_ gas pressure can supply rich carbon species for the thicker graphitic shell of the Co–TiC@C NCs.

### 3.2 Electromagnetic properties

**Fig. 6:**
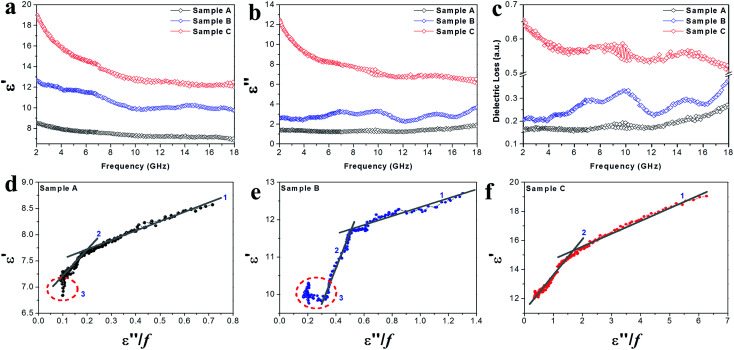
(a) The complex permittivity real part *ε*′, (b) complex permittivity imaginary part *ε*′′, (c) dielectric loss and (d–f) plots of *ε*′ *vs.* (*ε′′*/*f*) of the Co–TiC NCs.

**Fig. 7:**
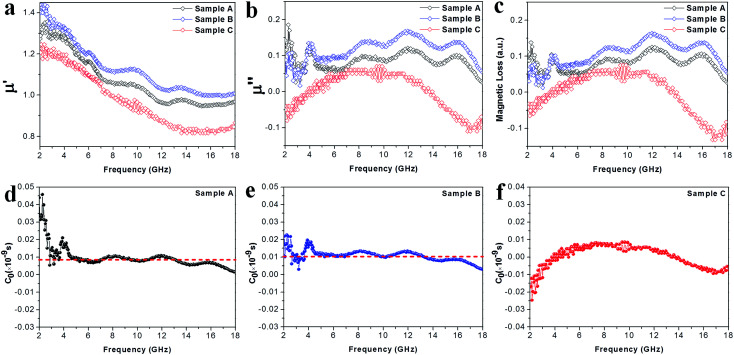
(a) Real parts *μ*′ of the complex permeabilities of the samples. (b) Imaginary parts *μ*′′ of the complex permeabilities of the samples. (c) The magnetic loss factors of the samples. (d–f) The plots of *C*_0_ (*C*_0_ = *μ*′′/(*μ*′)^2^*f* ∝ *σd*^2^) *vs.* frequency for samples A–C, respectively.

The EM parameters (relative complex permittivity (*ε*_r_ = *ε*′ − i*ε*′′) and relative complex permeability (*μ*_r_ = *μ*′ − i*μ*′′)) of the Co–TiC@C NCs samples were measured at the room temperature, as shown in Fig. 6 and 7. As it is well known that the real parts (*ε*′, *μ*′) of the EM parameters represent the storage capability of the dielectric/magnetic energy and the imaginary parts (*ε*′′, *μ*′′) represent the loss capability of the dielectric/magnetic energy.^10,29^Fig. 6a and b present the frequency dependency of the dielectric parameters (*ε*′, *ε*′′) observed for the three samples A–C. A characteristic dielectric dispersion phenomenon^30^ is presented in the Co–TiC@C NCs, *i.e.* the values of *ε*′ decrease from 8.57, 12.7 and 19.05 to 6.89, 9.76 and 12.03, for samples A–C, respectively. The imaginary part *ε*′′ of sample A was retained almost constant across the whole frequency range and for sample B, a slight fluctuation of *ε*′′ between 1.23 and 1.91 was observed, which becomes a sharp decrease in sample C from 12.52 to 6.07. As verified above, the thickness of the graphitic shell increased from sample A to C and these graphitic layers were considered to be the main dielectric contribution of the Co–TiC@C NCs. Fig. 6c has clearly shown this dielectric effect of the carbonaceous shell through the frequency dependence of dielectric loss factors, among them the thickest graphitic shell of sample C is surely the most dielectric contributor.

Generally speaking, the dielectric losses mainly originate from the electronic polarization, the ionic polarization and the electric dipolar polarization, *etc.* in which the two formers usually occur in a much higher frequency region (10^3^ to 10^6^ GHz). Thus, the dielectric loss reported herein mainly arises from the electric dipolar polarization. During the polarization process, large amounts of EM energy irreversibly transforms to Joule thermal energy and leads to microwave attenuation, which is usually described by the Debye relaxation equation as follows:^10^2
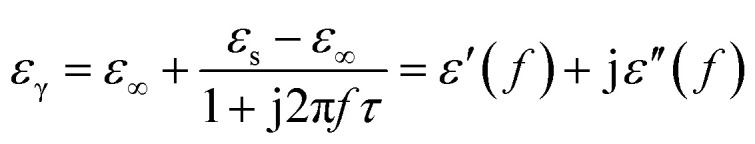
where *τ* is the relaxation time and *ε*_s_ and *ε*_∞_ are the stationary and optical dielectric constant, respectively. From eqn (2), it can be deduced that:3
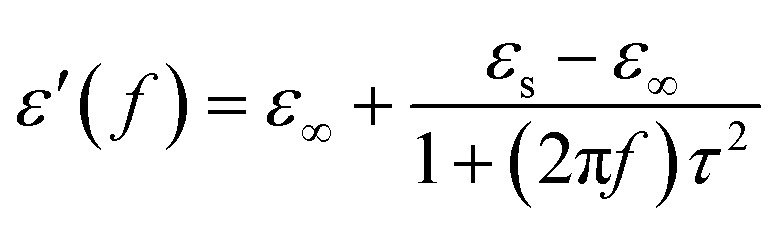
4
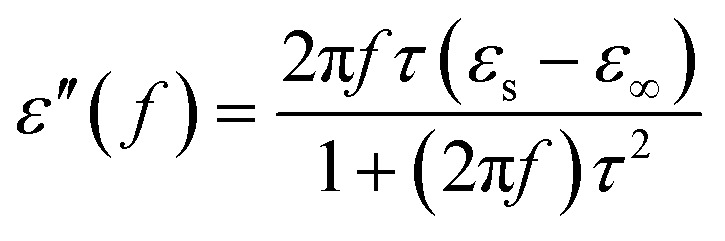
5
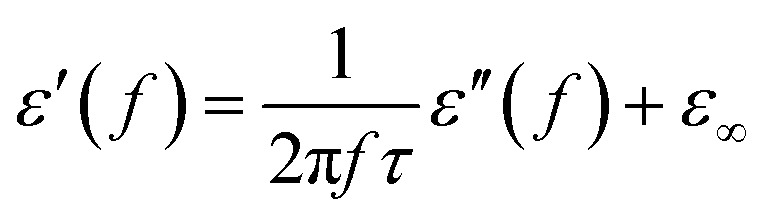


Arranging eqn (3) and (4) yields eqn (5), in which the plot of *ε*′ of *versus* (*ε*′′/*f*) will be linear if the dielectric loss comes from an individual dipolar polarization. Such plots have been carried out on samples A–C and are shown in Fig. 6d–f, respectively. It is suggested that an equivalent electric dipole model will be constructed for the single Co–TiC@C NCs at their different locations and interfaces such as: (1) a large number of defects in the graphitic shell probably hamper the migration of electric charges, which results in the formation of electric dipoles. It is considered that the π electrons embedded in the defective graphitic shells are localized so that they can't act well as the conductive electrons,^24^ most of them accumulate in an asymmetrical charge distribution^31^ and contribute to the dielectric polarization. (2) The heterogeneous interface between the graphitic shell and the Co–TiC twin core may induce an interfacial polarization,^32^ analogous to the electric dipoles. It is reasonable that the quite different dielectric substances in the graphitic shell and TiC core can promote charge accumulation at the interfaces. (3) The Co–TiC twin core may bring electric dipolar polarization itself. Such a polarization will be weak due to the double-core encapsulated inside the graphitic layers. As shown in the circles of Fig. 6d and e, the Co–TiC twin cores of samples A and B behave a weak response to EM waves in the high frequency range, which further become degraded and disappear for sample C.

Apart from the dielectric properties, the magnetic response is another loss factor caused in the Co–TiC@C NCs. As shown in the Fig. 7a, the *μ*′ values decrease upon increasing the frequency from 1.35 to 0.95, 1.44 to 1.01 and 1.24 to 0.84 in the frequency range of 2–18 GHz for sample A, B and C, respectively. Sample A and B shown a similar trend and multi-resonance peaks in *μ*′ and *μ*′′ *vs.* frequency, indicating that both of them have comparable magnetic and core–shell structures. In particular, it is observed that the *μ*′′ values of sample C are negative in the ranges of 2–4 GHz and 13.7–18 GHz. It is thought that the negative *μ*′′ values denote the magnetic energy radiated outwards.^32^ According to the Maxwell equations, the motion of the charges in an EM field will produce an ac electric field and induce an alternating magnetic field.^33^ Such an induced magnetic field possibly results in the negative values of *μ*′′ in certain structures, such as carbon nanotubes and graphenes.^34,35^ Here, the negative *μ*′′ values of sample C may be attributed to the well-developed graphitic layers.

To uncover the general magnetic losses among the three samples, Fig. 7c illustrates their magnetic loss factors *vs.* frequency. It was found that sample B has the highest magnetic loss, although its dielectric loss stays at the middle level among all the samples (Fig. 6c). Generally, the magnetic loss is thought to arise from hysteresis loss, domain-wall resonance, eddy current loss, natural resonance and exchange resonance of the nanostructure materials.^36^ The hysteresis loss can be negligible in a weakly applied field, which is mainly caused by the time lags of the magnetization vector behind the external EM field vector.^37^ The grain size of the Co core of Co–TiC@C NCs was less than the calculated critical size of a single magnetic domain (55 nm), thus the domain wall resonance was ignored in this work.^38^ In addition, the existence of high defective graphitic shells on the Co–TiC@C NCs may probably induce an eddy current loss, which can be evaluated using eqn (6):

6 *μ*′′ ≈ 2π*μ*_0_(*μ*′)^2^*σd*^2^*f*/3

where *μ*_0_ is the permeability in a vacuum, *d* is the particle's diameter and *σ* is the electric conductivity. Eqn (6) is further arranged to *C*_0_ in eqn (7):7
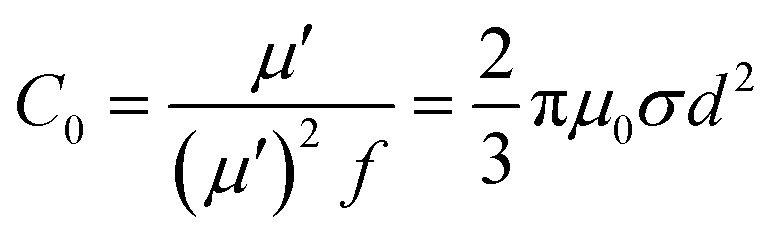


If the magnetic loss is only caused by the eddy current loss, the values of *C*_0_ are constant with the varying frequency. Fig. 7d–f shows the curves of *C*_0_*vs.* frequency observed for samples A–C, respectively. Apparently, the *C*_0_ values of samples A and B remain virtually constant with slight fluctuations from 5 to 18 GHz, indicating that there exists an eddy current loss in the two samples. Whereas the *C*_0_ values of sample C can be considered as continuously changing into a semicircle shape upon increasing the frequency, implying the contribution of eddy current loss was negligible. It is proposed that the eddy current may be suppressed due to the thickest graphitic shells in sample C, but is inevitable in samples A and B. In addition to the eddy current loss, natural resonance and exchange resonance also promote the magnetic loss for EM waves. The natural resonance frequency can be calculated according to the following equations:^39,40^

8 *H*_eff_ = 4|*K*|/(3*μ*_0_*M*_s_)

9 2π*f*_γ_ = *γ* × *H*_eff_

where *K* is the magneto-crystalline anisotropy constant, about 5.2 × 10^6^ erg cm^−3^ for bulk cobalt,^41^*γ* is the gyromagnetic ratio, about 2.92 × 10^−3^ GHz Oe^−1^,^42^ and *μ*_0_ and *M*_s_ are the universal value of permeability in free space and the saturation magnetization. Fig. S1† shows that the measured saturation magnetization of samples A–C was 104.5, 99.8 and 88.3 emu g^−1^, respectively. Based on eqn (8) and (9), the calculated *f*_γ_ were estimated to be 3.5, 3.6 and 4.1 GHz for samples A–C, respectively. The sharp peak appearing at about 4 GHz (Fig. 7b) was attributed to the natural resonance of samples A and B.^43,44^ Due to the particularity of sample C with its thicker graphitic shell and magnetic Co core, its natural resonance frequency will be greatly influenced by the effective anisotropic field *H*_eff_, which was determined by the saturation magnetization, crystallographic characteristic or morphology, *etc.* Accordingly, the resonance peak of sample C becomes broader and shifts to high frequency. Moreover, the three peaks appearing at 8.7, 12.0 and 16.0 GHz for samples A and B should belong to the exchanged resonances, according to Aharoni's theory.^45^

**Fig. 8:**
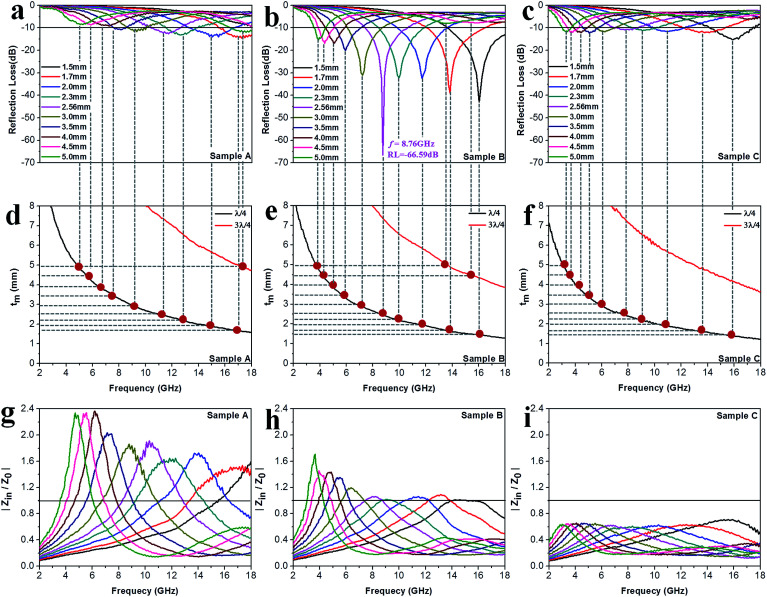
Microwave absorption performance. (a–c) Calculated reflection loss values using samples A–C as the filler, respectively. (d–f) Plots of *λ*/4 and 3*λ*/4 *vs.* the frequency of minimum RL. (g–i) The corresponding relationship between |*Z*_in_/*Z*_0_| and frequency.

To gain an insight into the microwave absorption ability of the Co–TiC@C NCs, using the measured EM parameters, the reflection losses RL (dB) of three samples were quantitatively estimated in transmission line theory using eqn (10):10
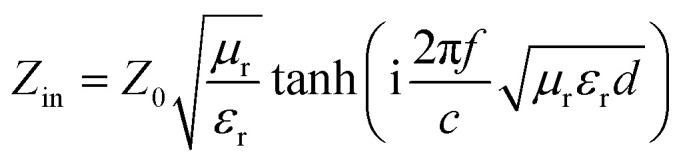
where *Z*_0_ (377 Ω) is the impedance of free space and *Z*_in_ refers to the input characteristic impedance of a metal-backed microwave absorbing layer. *Z*_in_ is given by eqn (11):^46^11
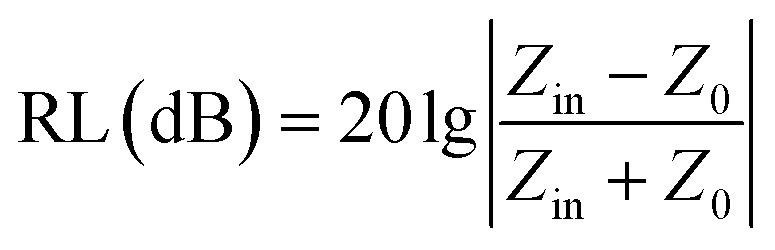
where *d* is the thickness of the absorber. Fig. 8a–c exhibit the calculated RL curves *vs.* frequency with the incremental thickness of samples A–C, respectively. It is shown that sample A and C reach the minimum RL around −10 dB in whole frequency range in spite of any thickness of the absorbents. Moreover, sample B displays excellent EM wave absorption with the strongest reflection loss up to −66.59 dB at 8.76 GHz with a thickness of 2.56 mm, and can even keep a minimum reflection loss below −10 dB covering a wide bandwidth of 14.4 GHz from 3.6 to 18 GHz in the thickness range of 1.5 to 5.0 mm. It is interesting that although the three samples possess analogous Co–TiC@C core@shell structures, they display quite different abilities towards absorbing EM waves. The excellent balance between the dielectric and magnetic losses has been built in sample B using an appropriate thickness of the graphitic shell and a compatible double-core Co–TiC. This means the sample B opens an entrance for incident microwaves and consumes it to the utmost extent. In order to clarify the impedance matching between the entity of the composite and air, it is calculated using eqn (12):12
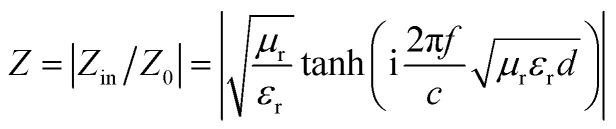


As we know, when *Z*_in_ equals *Z*_0_, the reflection of EM waves at the air/absorber interface can be zero. Nevertheless, such idealized impedance matching is difficult to be realized in practical situations. An effective control over the dielectric and magnetic components of the Co–TiC@C NCs is necessary, so that *Z*_in_ is close to *Z*_0_. The frequency dependences of *Z* for the three samples are shown in Fig. 8g–i. By comparing the three samples, the *Z* values of sample B generally distribute around 1.0, which means that it has a better impedance matching than the other two samples and more EM waves can propagate into the absorber to be attenuated.

In addition to the dielectric/magnetic losses from the absorbent itself, the EM waves can also be attenuated *via* a “geometric effect”, which is called the *λ*/4 matching model. If the absorber thickness *t*_m_ at the peak frequency *f* meets with eqn (13):13
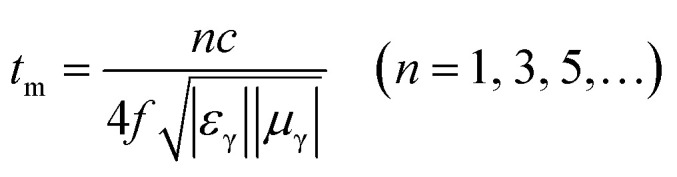
where |*ε*_r_| and |*μ*_r_| are the moduli of *ε*_r_ and *μ*_r_, the incident and reflected waves in the absorber are out of phase by 180° and will give rise to a destructive interference at the interfaces between the air space and the composite absorber. Fig. 8d–f show the plots of *λ*/4 and 3*λ*/4 *versus* the frequency of minimum RL and the absorber's thickness for samples A–C, respectively. The red dots signify the experimental matching thickness at the minimum RL values at the matched frequencies, and the two types of curves (the black ones: *λ*/4 lines, the red ones: 3*λ*/4 lines) denote the simulative thickness using the *λ*/4 matching model at the same frequency range of 2–18 GHz. Apparently, the experimental values at the given thickness are coincident with the simulative values, which means that there exists an interference loss in these three samples. On the other hand, it is also can be seen that more EM waves can be dissipated by the interference in sample B when compared with samples A and C. Moreover, it is worth noting that the red dots present a tendency of moving to lower frequencies from sample A to sample C, which maybe related to the increasing *ε*_r_ values upon the enhanced pressure ratio of CH_4_/Ar.

## 4. Conclusion

In summary, we have, for the first time, demonstrated that multiple-phase carbon coated Co–TiC twin cores NCs can be conveniently synthesized *via* an *in situ* arc-discharge plasma method. CH_4_ acts as the reactant gas and provides the carbon sources to form the TiC and graphitic shell. By accurately changing the gas pressure ratios of CH_4_ to Ar, it can effectively modulate the microstructure, phase proportion and optimize the EM properties of the as-prepared NCs. Due to the appropriate complementary between the magnetic loss of Co and the dielectric loss of TiC/C, the minimum reflection loss can achieve −66.59 dB at 8.76 GHz with a low thickness of 2.56 mm and the bandwidth of RL ≤ −10 dB reaches 14.4 GHz, covering from 3.6 to 18 GHz. It is anticipated that this work opens the door towards the synthesis of other multiple-phase system including magnetic/dielectric loss types of microwave absorbents with enhanced microwave absorption performance in a broad frequency range.

## Conflicts of interest

There are no conflicts to declare.

## Supplementary Material

RA-008-C8RA00040A-s001
