# Supplementary material for: Novel nanocapsules with Co–TiC twin cores and regulable graphitic shells for superior electromagnetic wave absorption
Source: RSC Adv. 2018 Feb 8;8(12):6397–405. doi: 10.1039/c8ra00040a (PMC9078270; doi:10.1039/c8ra00040a)
Supplement: RA-008-C8RA00040A-s001 [file RA-008-C8RA00040A-s001.pdf]

Electronic Supplementary Material (ESI) for RSC Advances.
This journal is © The Royal Society of Chemistry 2018

Electronic Supplementary Information

Novel nanocapsules with Co-TiC twin cores and regulable graphitic shells for superior electromagnetic wave absorption

Yuanliang Zhou,[a] Javid Muhammad,[a] Xuefeng Zhang,[b] Dongxing Wang,[a] Yuping Duan,[a] Xinglong Dong*[a] and Zhidong Zhang*[c]

[a] *Key Laboratory of Materials Modification by Laser, Ion, and Electron Beams, School of Materials Science and Engineering, Dalian University of Technology, Liaoning, 116024, PR China*

[b] *Key Laboratory for Anisotropy and Texture of Materials (MOE), School of Materials and Engineering, Northeastern University, Shenyang, 110819, PR China*

[c] *Shenyang National Laboratory for Materials Science, Institute of Metal Research, Chinese Academy of Sciences, Shenyang, Liaoning, 110015, PR China*

Table S1. Calculated weight fraction of Co-TiC in the samples A, B and C, respectively.

Samples	Area integral intensity of the strongest XRD peak		Calculated phase fraction in the cores (wt%)	
	TiC (200)	Co (111)	TiC	Co
A	11.76	110.57	15.74	84.26
B	12	97.83	17.73	82.27
C	23.63	95.77	30.2	69.8

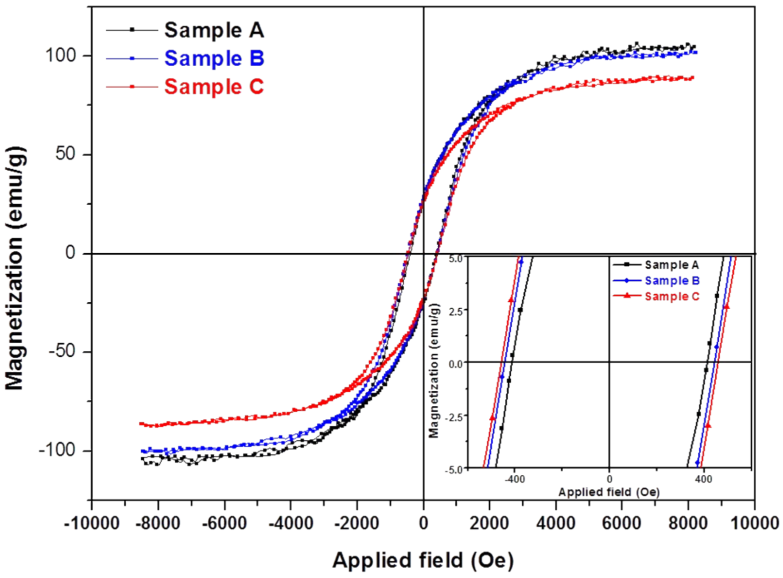

Figure S1. Room temperature hysteresis loops of the Co-TiC@C NCs samples A-C.
